# *TBX2* affects proliferation, apoptosis and cholesterol generation by regulating mitochondrial function and autophagy in bovine cumulus cell

**DOI:** 10.1002/vms3.1009

**Published:** 2022-11-29

**Authors:** Sheng‐Peng Li, Hao Jiang, Zi‐Bin Liu, Wen‐Jie Yu, Xiao‐Shi Cai, Chang Liu, Wen‐Yin Xie, Fu‐shi Quan, Wei Gao, Nam‐Hyung Kim, Bao Yuan, Cheng‐Zhen Chen, Jia‐Bao Zhang

**Affiliations:** ^1^ Department of Laboratory Animals Jilin Provincial Key Laboratory of Animal Model Jilin University Changchun Jilin China; ^2^ School of Grains Jilin Business and Technology College Changchun Jilin China; ^3^ School of Biotechnology and Healthcare Wuyi University Jiangmen Guangdong China

**Keywords:** autophagy, cumulus cells, physiological function, reactive oxygen species, T‐box transcription factor 2

## Abstract

**Background:**

T‐box transcription factor 2 (*TBX2*) is a member of T‐box gene family whose members are highly conserved in evolution and encoding genes and are involved in the regulation of developmental processes. The encoding genes play an important role in growth and development. Although *TBX2* has been widely studied in cancer cell growth and development, its biological functions in bovine cumulus cells remain unclear.

**Objectives:**

This study aimed to investigate the regulatory effects of *TBX2* in bovine cumulus cells.

**Methods:**

*TBX2* gene was knockdown with siRNA to clarify the function in cellular physiological processes. Cell proliferation and cycle changes were determined by xCELLigence cell function analyzer and flow cytometry. Mitochondrial membrane potential and autophagy were detected by fluorescent dye staining and immunofluorescence techniques. Western blot and quantitative real‐time reverse transcription polymerase chain reaction (qRT‐PCR) were used to detect the expression changes of proliferation and autophagy‐related proteins. Aadenosine triphosphate (ATP) production, glucose metabolism, and cholesterol synthesis of cumulus cells were measured by optical density and chemiluminescence analysis.

**Results:**

After inhibition of *TBX2*, the cell cycle was disrupted. The levels of apoptosis, ratio of light chain 3 beta II/I, and reactive oxygen species were increased. The proliferation, expansion ability, ATP production, and the amount of cholesterol secreted by cumulus cells were significantly decreased.

**Conclusions:**

*TBX2* plays important roles in regulating the cells’ proliferation, expansion, apoptosis, and autophagy; maintaining the mitochondrial function and cholesterol generation of bovine cumulus cells.

## Graphical abstract

TBX2 has regulatory roles in regulating the cells proliferation, expansion, apoptosis, autophagy, maintaining the mitochondrial function, and cholesterol generation of bovine cumulus cells.

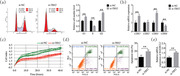

## 1 INTRODUCTION

Improving the quality of oocytes is important for animal husbandry and human fertility (Xu et al., 2018) because the quality of oocytes is the primary factor affecting the fertilisation and breeding of healthy offspring (Keefe et al., 2015). As a subgroup of granular cells, cumulus cells play important role in the nutrition and maturation of oocytes (Dumesic et al., 2015). Oocytes are coupled to surrounding cumulus cells through interstitial junctions (Petro et al., 2012), and this highly specific membrane junction forms the regulatory system that mediates the intercellular transfer of metabolites and regulatory molecules (Lin et al., 2016). Under normal physiological conditions, oocytes generate many factors that regulate cumulus cells, and cumulus cells provide nutrients for oocyte growth through an intracellular exchange; these processes are interdependent but closely related (Gilchrist et al., 2008). In addition, oocytes, under pathological conditions, protect themselves against oxidative stress through antioxidant‐scavenging enzymatic (e.g., involving catalase and glutathione peroxidase) and nonenzymatic (e.g., involving ascorbic acid and reduced glutathione) networks provided by the surrounding cumulus cells (Cetica et al., 1999, 2001; Shaeib et al., 2016).

As intracellular energy factories, mitochondria are the main producers of intracellular reactive oxygen species (ROS) (Oyewole & Birch‐Machin, 2015), which are involved in processes such as cell differentiation, cell signal transmission, cell apoptosis, and the regulation of cell growth and the cell cycle. When excessive production of ROS occurs, the accumulation of oxidants exceeds the cell's ability to clear them, and the oxidation system and antioxidant system become unbalanced. A sharp increase in ROS will lead to oxidative stress, causing cell damage and apoptosis (Scherz‐Shouval et al., 2007). In the process of cell apoptosis, the ratio of BAX/BCL2 (Kulsoom et al., 2018; Lossi et al., 2018) directly determines the degree of opening of various channels in the mitochondrial outer membrane, and BAX and BCL2 represent a regulatory hub of cell apoptosis, while *Caspase 3* usually co‐regulates apoptosis with BAX and BCL2 (Zhao et al., 2018). In addition, preferential autophagy of damaged or excess organelles such as peroxisomes, the endoplasmic reticulum and mitochondria can occur in response to ROS (Scherz‐Shouval & Elazar, 2007). ROS‐mediated autophagy and apoptosis of cumulus cells will affect their secretory functions, thus potentially affecting the development and quality of oocytes (Adriaenssens et al., 2010; Fu, Chen, Wang, et al., 2019; Huang & Wells, 2010).

T‐box gene family is a phylogenetically conserved family of genes that share a common DNA‐binding domain (Chapman et al., 1996) and is important in the regulation of body development. Research has shown that T‐box transcription factor 2 (*TBX2*) functions as a transcriptional repressor during germ layer formation, and this activity is mediated in part through repression of target genes stimulated in the mesendoderm by transactivating T‐box proteins (Teegala et al., 2018). In addition, *TBX2*, as a transcription factor, is involved in embryonic development and cell cycle regulation and inhibits the cycle regulation factors *p21* and *p14* to make cells resist senescence (Abrahams et al., 2010; Jacobs et al., 2000; Peres et al., 2010). Recent research also showed that after the *TBX2* gene was knocked down, cell proliferation and invasion were significantly decreased; after *TBX2* was overexpressed, cyclin E and the phosphorylated extracellular signal‐regulated kinase levels were upregulated (Liu et al., 2019).

Although *TBX2* has been extensively studied in cancer cells (Crawford et al., 2019), its biological functions in bovine cumulus cells remain unclear. This study investigated the effects of *TBX2* on ROS levels, mitochondrial function, and cell proliferation in cumulus cells by inhibiting the expression of *TBX2*. The results will provide a new basis for understanding the biological roles of *TBX2* as well as cumulus cells.

## 2 MATERIALS AND METHODS

The chemicals and reagents that we used in the experiment were bought from Sigma–Aldrich except expressly stated elsewise in the article.

### 2.1 Isolation and culture of bovine cumulus cells

Bovine ovaries without corpus luteum were collected from a local slaughterhouse and transported to the laboratory at 35°C in saline supplemented with 75 mg/ml penicillin G and 50 mg/ml streptomycin sulfate. Cumulus–oocyte complexes (COCs) were aspirated from follicles 3 to 8 mm in diameter using a 10‐ml syringe with an 18‐gauge needle. Then, COCs surrounded by a minimum of three cumulus cells were selected and washed three times in Tyrode's lactate 2‐[4‐(2‐hydroxyethyl)piperazin‐1‐yl]ethanesulfonic acid supplemented with 0.1% (w/v) polyvinyl alcohol and gentamycin (0.05 g/l). Subsequently, COCs were dissociated with 1% hyaluronidase to separate cumulus cells and oocytes. After removing the oocytes, the cumulus cells were centrifuged at 500 × *g* for 5 min and seeded into the 25‐cm^2^ culture plate (Nest Biotechnology) with cell culture medium including Dulbecco’s Modified Eagle Medium with Ham’s F12 (DMEM/F12, Gibco), 1% penicillin and streptomycin (HyClone), and 10% fetal bovine serum (FBS, Biological Industries) and cultured at 38.5°C in 5% CO_2_.

### 2.2 siRNA treatment

A total of 1 × 10^5^ cells were seeded into six‐well plates (Nest Biotechnology) containing cell culture medium for 24 h. Then, *TBX2*‐specific siRNA (si‐*TBX2*) and negative control scrambled siRNA (si‐NC; no effects on known mammalian gene expression) (GenePharma Co. Ltd.) were administered with RiboFECTCP (Guangzhou RiboBio Co. Ltd.) reagent into cells at a confluence of approximately 70% according to the manufacturers’ instructions and our previous study (Fu, Chen, Wang, et al., 2019). Then, the cells were incubated for 48 h at 38.5°C in a 5% CO_2_ incubator without changing the culture medium. The specific sequences of the siRNAs are shown in Supplementary Table S1.

### 2.3 Cell cycle assay

In brief, 1 × 10^5^ cells were seeded in six‐well culture plates. After siRNA treatment for 48 h, the cell cycle distribution was determined using a Cell Cycle and Apoptosis Analysis Kit (Beyotime) and a flow cytometer (Beckman Coulter) according to the manufacturers’ instructions. The data were processed by using MODFIT software (Verity Software House).

### 2.4 Quantitative real‐time reverse transcription polymerase chain reaction (qRT‐PCR)

Total RNA was extracted using Tripure Isolation Reagent (Roche). cDNA was synthesised from the extracted RNA with a reverse transcription kit (Tiangen) according to the instructions. Gene expression was quantified with a Mastercycler ep realplex system (Eppendorf) and the 2^−ΔΔCt^ method with *β‐actin* as the reference gene using the following protocol: 95°C for 3 min; 40 cycles at 95°C for 30 s, 60°C for 30 s and 72°C for 30 s. All primers used are listed in Supplementary Table S1.

### 2.5 Cell proliferation assay

The cell proliferation was assayed using xCELLigence system (Roche Applied Science and ACEA Biosciences) as described previously with some modifications (Bird & Kirstein, 2009; Urcan et al., 2010). In brief, 50 μl of cell culture media with si‐*TBX2* or si‐NC at room temperature was added into each well of E‐plate 16. Then, the E‐plate 16 was connected to the system and checked in the cell culture incubator for proper electrical contacts, and the background impedance was measured. Meanwhile, the cells were resuspended in a cell culture medium with siRNAs. Hundred microlitres of each cell suspension containing 5 × 10^3^ cells was added to the 50 μl medium containing si‐*TBX2* or si‐NC on E‐plate 16 in order to determine the optimum cell concentration. After 30 min incubation at room temperature, E‐plate 16 was placed into the cell culture incubator. Finally, cell proliferation was monitored every 30 min for a period of up to 40 h via the incorporated sensor electrode arrays of the E‐Plate 16. The electrical impedance was measured by the Real Time CelI AnaIysis (RTCA)‐integrated software of the xCELLigence system as a dimensionless parameter termed cell index.

### 2.6 Apoptosis detection

The apoptosis of cumulus cells was detected according to the instructions of the Annexin V‐Fluorescein isothiocyanate (FITC) Apoptosis Analysis Kit (Tianjin Sungene Biotech Co. Ltd.). In brief, cells were treated with trypsin and washed twice with phosphate‐buffered saline (PBS). Then, the cells were centrifuged at 800 × *g* for 5 min at 4°C and three times. Then, 100 μl of PBS was added to each centrifuge tube, followed by incubation for 15 min with 5 μl of FITC‐labeled Annexin V solution and 5 μl of propidium iodide (20 μg/ml) in the dark. Apoptotic and viable cells were distinguished by staining with propidium iodide and FITC‐labeled annexin V, respectively. The cell samples were then analysed using a flow cytometer (Beckman Coulter).

### 2.7 Mitochondrial membrane potential (MMP) measurement

Briefly, the high‐transparency cell culture slides (Nest Biotechnology; Cat. No. #801009) were placed into the six‐well plate before the cells were cultured. After siRNA treatment, the culture medium was removed, and the cell slides were washed twice with PBS. Then, 1 ml of PBS containing 2 μM 5,5',6,6'‐tetrachloro‐1,1',3,3'‐tetraethylbenzimidazolylcarbocyanineiodide (JC‐1; Beyotime) dye staining working solution was added to each of the wells, and the plates were incubated at 38.5°C for 30 min. After incubation, the culture slides were washed twice with PBS and mounted onto glass slides. The red and green fluorescence intensities were observed with a fluorescence microscope (Olympus). The red and green fluorescence and the ratio between the average optical densities in each sample were analysed by Image‐Pro Plus software (Media Cybernetics) according to its manual (https://www.mediacy.com/images/ipplus/IPPStartUp.pdf). The relative fluorescence intensity level was measured as the average fluorescence intensity of individual cells (JC‐1_red_/JC‐1_green_) from three independent experiments with three randomly selected observation images.

### 2.8 Determination of ROS levels

We first detected the level of ROS by microscopic fluorescence imaging. In brief, the cell culture slides were placed in the six‐well plate before the cells were cultured. After siRNA treatments, the culture medium was removed. Subsequently, 1 ml DMEM/F12 culture medium containing 10 μM 2′,7′‐dichlorodihydrofluorescein diacetate (DCFH‐DA, Beyotime) was added to each well of the six‐well plate. After incubation for 30 min in a 38.5°C incubator, the culture slides were washed three times with PBS and mounted onto glass slides. Finally, the fluorescence intensity of the cells was observed by a fluorescence microscope (Olympus). The relative fluorescence intensity level was measured as the average fluorescence intensity of individual cells from three independent experiments in three randomly selected fields. Meanwhile, the changes in the ROS level were also detected with a ROS Assay Kit (Beyotime) according to the manuals by the flow cytometry as we described (Fu, Chen, Wang, et al., 2019).

### 2.9 Immunofluorescence detection

The cell culture slides were placed into the six‐well plate before the cells were cultured. After siRNA treatment, the culture medium was removed, and the cells were washed twice with PBS. After being fixed with 4% paraformaldehyde for 30 min and being washed three times with PBS, the cells were permeabilised with 0.3% Triton X‐100 in PBS for 30 min and blocked in an incubator at 38.5°C for 1 h in PBS containing 3% bovine serum albumin (BSA). Then, the cells were incubated at 4°C with primary anti‐light chain 3 beta (LC3B) antibody (Abcam, #ab48394, 1:200) overnight. Subsequently, after being washed three times with PBS, the cells were incubated with Alexa Fluor 488‐conjugated secondary antibody (Abcam, #ab150077, 1:1000) at 38.5°C for 1 h, washed three times and stained with Hoechst 33342 for 10 min. Finally, after being washed three times with PBS, the culture slides were mounted onto glass slides. A Zeiss LSM 510 confocal microscope (Carl Zeiss) and Image‐Pro Plus software were used to detect and analyse the images, respectively. The relative fluorescence intensity level was measured as the average fluorescence intensity of individual cells from three independent experiments in three randomly selected fields.

### 2.10 Cell expansion detection

COCs were cultured in TCM199 with 20 mM bicarbonate containing 0.2 mM sodium pyruvate, 0.5 μg/ml follicle‐stimulating hormone, 5.0 μg/ml luteinizing hormone, 10 μg/ml gentamicin, 10% FBS supplemented with si‐NC and si‐TBX2 and covered with mineral oil at 38.5°C in a humidified atmosphere of 5% CO_2_ in the air. Cell expansion was detected at 0 and 24 h through optical microscopy (Motic). Image‐Pro Plus software was used to calculate the average area of the expansion.

### 2.11 Cholesterol and lactic acid detection

Briefly, the cells were cultured into the six‐well plate. After siRNA treatment, the culture medium was removed, and the cells were washed twice with PBS. Cholesterol and lactic acid were detected according to the instructions from total cholesterol assay kit and lactate dehydrogenase activity quantitative assay kit (Applygen). Finally, Multi‐scan Spectrum was used to detect the optical density value.

### 2.12 Western blotting analysis

The cells were cultured and treated with si‐*TBX2* or si‐NC in six‐well plates as described above. After removing the culture medium, cell lysis buffer containing protease inhibitors (Beyotime) was added to the well, and the samples were ultrasonicated for 60 s (three times per second) with an ultrasound transducer. After sonication, the cell sample mixtures were harvested and were placed on ice for 30 min. Then, the mixtures were centrifuged for 60 min at 13,000 × *g*, and the protein‐containing supernatant was collected. Subsequently, protein samples were separated by sodium dodecyl sulfate–polyacrylamide gel electrophoresis and then transferred to a 0.45‐μm polyvinylidene fluoride membrane (Millipore). Next, the membrane was transferred to 5% BSA blocking solution and placed on an oscillator for 2 h at room temperature and then incubated with specific primary antibodies (anti‐LC3B, Abcam, #ab48394, 1:1,000; anti‐β‐actin, Cell Signaling Technology, #4970, 1:1,000) overnight at 4°C. After being washed with tris‐buffered saline with 0.1% Tween 20 detergent, the membranes were incubated with horseradish peroxidase‐conjugated secondary antibodies (Cell Signaling Technology, #7074, 1:5000) at 37°C for 1 h. The membranes were then incubated in enhanced chemiluminescence reagents (Thermo), and images were captured by a Tanon 5200 chemiluminescence imaging analyzer (Tanon Science & Technology Co. Ltd.). Finally, grayscale analysis was performed using Image‐Pro Plus software.

### 2.13 Adenosine triphosphate (ATP)‐level detection

The ATP level was determined by an ATP assay kit (Beyotime) according to the manufacturer's protocol. Briefly, after the culture of the siRNA treatment, the cells were collected and lysed with lysis buffer. Then, the cell lysates were centrifuged at 12,000 × *g* at 4°C for 10 min. After that, the supernatant was obtained. Next, 100 μl of ATP working solution and 20 μl of supernatant were mixed well and added to a 96‐well plate (Nest Biotechnology). At last, the luminance of the mixture was quantified by an Infinite M200 microplate reader (Tecan).

### 2.14 Statistical analysis

The results were obtained from three repeated independent experiments and were expressed as means ± standard deviation. Data obtained from two groups were compared using the Student's *t* test. All statistical analyses were performed using SPSS version 22.0 (IBM) software. *P* < 0.05 and *P* < 0.01 were considered to indicate significant differences.

## 3 RESULTS

### 3.1 Inhibition of *TBX2* reduces cell proliferation and disrupts the cell cycle in bovine cumulus cells

**FIGURE 1:**
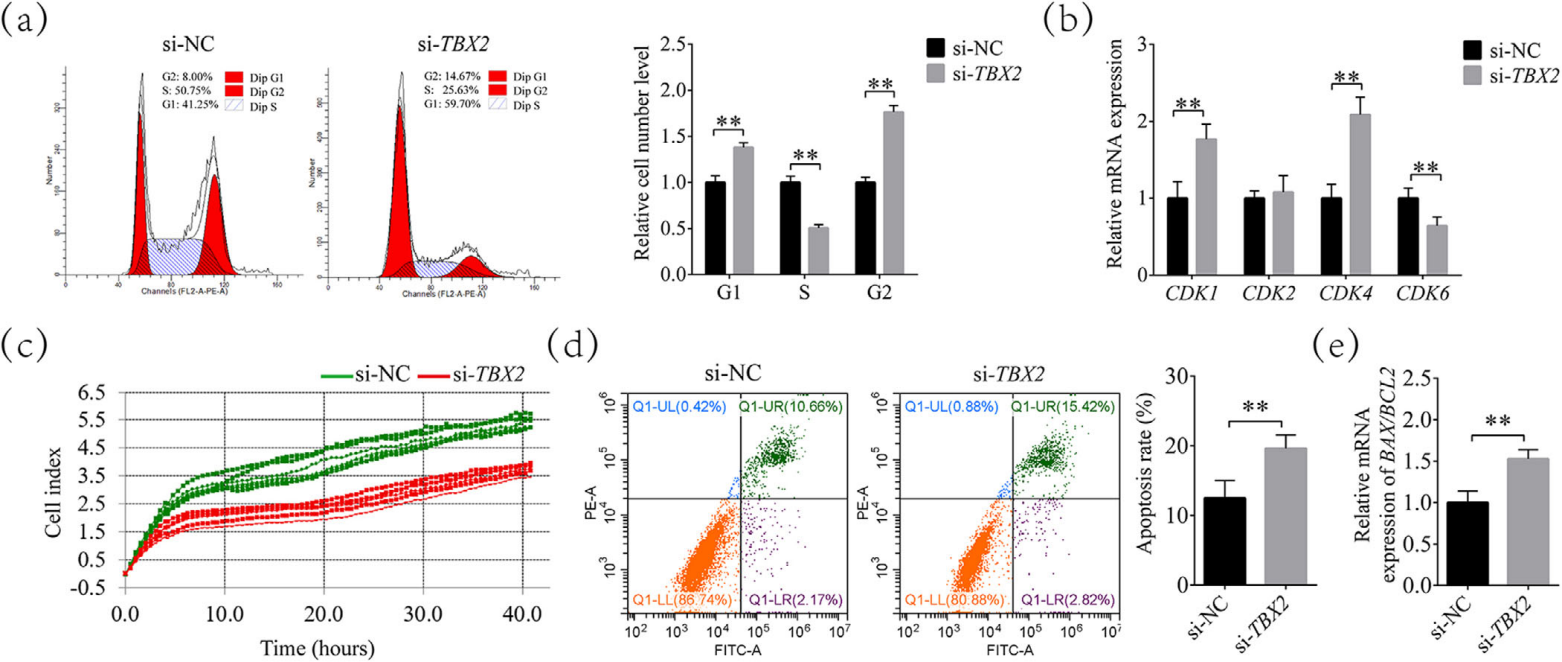
T‐box transcription factor 2 (*TBX2* )inhibition affects the cell cycle, proliferation, and apoptosis. (a) The percentage of cells in each phase of the cell cycle with or without *TBX2* inhibition. (b) Differential expression of *CDK1*, *CDK2*, *CDK4*, and *CDK6* in cumulus cell of negative control scrambled siRNA (si‐NC) and si‐*TBX2* groups. (c) Cell index in cumulus cell of si‐NC and si‐*TBX2* groups. (d) Percentage of apoptotic cells in cumulus cell of si‐NC and si‐*TBX2* groups. (e) Changes in *BAX/BCL2* mRNA levels ratio. Significant differences are represented with ** (*P* < 0.01).

After comparison, the most effective and stable siRNA (siRNA‐708) was selected for subsequent experiments (Supplementary Figure S1). As shown in Figure 1a, the cell cycle was significantly changed after *TBX2* inhibition, and the proportion of G1 phase cells in the *TBX2*‐inhibited group increased to 1.38 ± 0.05 times that in the control group (*P* < 0.01). The proportion of S phase cells decreased to 0.51 ± 0.04 times that in the control group (*P* < 0.01). The proportion of G2 cells increased to 1.76 ± 0.07 times that in the control group (*P* < 0.01). The expression levels of cyclin‐dependent kinase *(CDK)1* and *CDK4* genes increased to 1.77 ± 0.20 (*P* < 0.01) and 2.09 ± 0.23 times (*P* < 0.01) than in the control group, respectively. The *CDK6* gene was downregulated 0.64 ± 0.11 times (*P* < 0.01), while the expression of the *CDK2* gene had no significant change (*P* > 0.05, Figure 1b). In addition, the cell index in the *TBX2*‐inhibited group was lower than that of the control group after approximately 5 h, and this effect lasted for at least 40 h (Figure 1c). After *TBX2* inhibition, the apoptosis rate of cumulus cells increased from 12.48 ± 2.50% to 19.61 ± 1.95% (Figure 1d). Compared with the control group, the BCL2‐associated X (*BAX*)/B‐cell lymphoma 2 (*BCL2)* level in the *TBX2* inhibition group increased by 1.53 ± 0.11 times (Figure 1e).

### 3.2 *TBX2* inhibition leads to an increase in ROS accumulation in bovine cumulus cells

**FIGURE 2:**
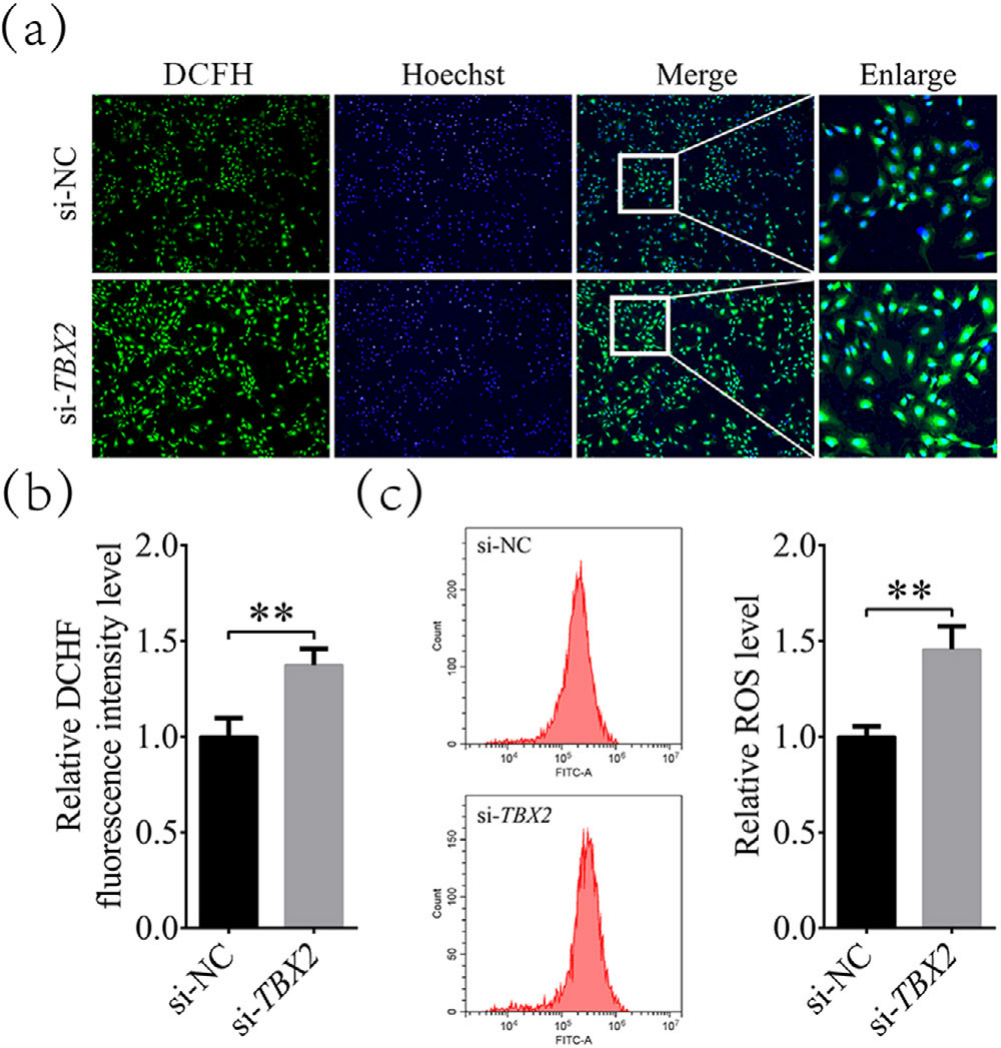
ROS levels changes in cumulus cell after inhibition of *TBX2*. (a) Representative fluorescence images of DCFH staining of cumulus cells. (b) Differences in DCFH levels between the si‐NC and *TBX2*‐inhibited groups. (c) ROS levels in the si‐NC and *TBX2*‐inhibited groups as detected by flow cytometry. Magnification = 100 ×. Significant differences are represented with ** (*P* < 0.01).

ROS are important factors that induce apoptosis in cells. Therefore, we tested whether *TBX2* regulates apoptosis by affecting intracellular ROS accumulation. As shown in Figures 2a and 2b, the DCFH fluorescence levels in the *TBX2* inhibition group were 1.37 ± 0.09‐fold higher than those in the si‐NC group. Furthermore, flow cytometry analysis (Figure 2c) indicated that the intracellular ROS levels of the *TBX2* inhibition group were significantly increased to 1.46 ± 0.12 times (*P* < 0.01) those of the si‐NC group. These suggested that inhibition of *TBX2* caused ROS accumulation in bovine cumulus cells.

**FIGURE 3:**
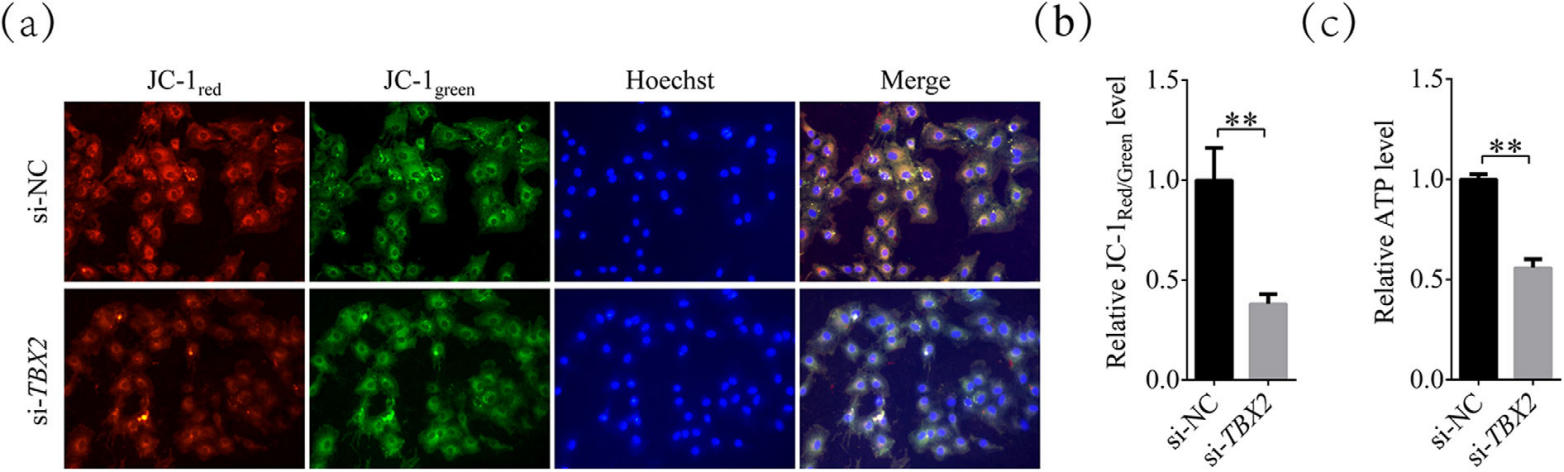
Changes in mitochondrial membrane potential and ATP levels after inhibition of *TBX2*. (a) Representative fluorescence images of JC‐1 staining in the *TBX2* inhibition group and si‐NC group. (b) Relative fluorescence intensity of JC‐1_red_/JC‐1_green_ in the si‐NC and *TBX2*‐inhibited groups. (c) Relative ATP levels in the si‐NC and *TBX2*‐inhibited groups. Magnification = 200 ×. Significant differences are represented with ** (*P* < 0.01).

### 3.3 Inhibition of *TBX2* disrupts mitochondrial function

As shown in Figure 3a,b, the ΔΨm of the average cell was calculated as a ratio of red fluorescence intensity (J‐aggregates; corresponding to activated mitochondria) to green fluorescence intensity (J‐monomers; corresponding to inactive mitochondria). The results showed that after inhibition of *TBX2*, the ΔΨm decreased to 0.38 ± 0.05 times, compared to those in the si‐NC group (*P* < 0.01). In addition, the ATP level (Figure 3c) in *TBX2*‐inhibited cumulus cells (downside) decreased to 0.56 ± 0.04 times, compared to those in the si‐NC group (upside; *P* < 0.01). These results suggested that the mitochondrial activity and function in cumulus cells decreased significantly after the inhibition of *TBX2*.

### 3.4 Inhibition of *TBX2* increases autophagy levels

**FIGURE 4:**
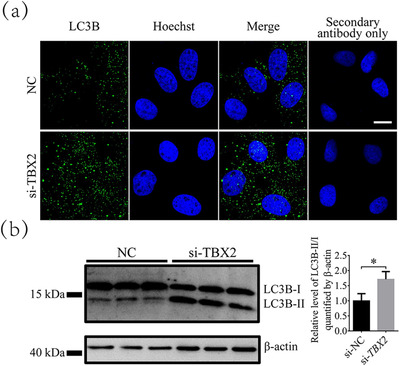
Autophagy level changes after inhibition of *TBX2*. (a) Representative light chain 3 beta (LC3B) staining images in the si‐NC and *TBX2*‐inhibited groups analysed by immunofluorescence. Scale bar = 10 μm. (b) Protein level changes of LC3B in the si‐NC and si‐*TBX2* groups. Significant differences are represented with * (*P* < 0.05).

Autophagy, which is usually measured by the levels of *LC3B*, maintains microenvironment stability in vivo, thereby reducing damage to the cells. After inhibition of *TBX2*, the immunofluorescence results showed a significant increase in the number of cytoplasmic *LC3B* dot (Figure 4a). The western blot results were also consistent with this finding. The relative level of LC3BII/I in the *TBX2*‐inhibited group was 1.71 ± 0.26‐fold higher than those in the si‐NC group (*P* < 0.05, Figure 4b).

### 3.5 *TBX2* inhibition prevents cumulus cell expansion

**FIGURE 5:**
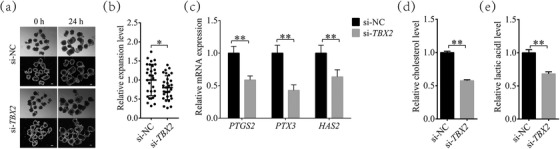
Inhibition of *TBX2* reduces cumulus cell expansion and inhibits cholesterol synthesis. (a) Representative images of cumulus–oocyte complex at 0 and 24 h with or without *TBX2* inhibition. Scale bar = 100 μm. (b) Compared with the si‐NC group, the *TBX2* inhibition group showed significantly lower cumulus cell expansion levels. Black dots represent the measurements distribution in si‐NC and *TBX2*‐inhibited groups. (c) Relative *PTGS2*, *PTX3*, and *HAS2* expression changes between the si‐NC and *TBX2*‐inhibited groups. (d) The relative level of cholesterol was decreased in the *TBX2*‐inhibited group compared with the si‐NC group. (e) The relative level of lactic acid was decreased in *TBX2*‐inhibited groups. Significant differences are represented with * (*P* < 0.05) and ** (*P* < 0.01).

The expansion level of cumulus cells is an important marker of oocyte maturation. As shown in Figure 5a,b, the relative expansion level of cumulus cells in the TBX2‐inhibited group was 0.80 ± 0.31‐fold lower than si‐NC group (*P* < 0.05) when oocytes matured. Meanwhile, the mRNA expression levels of the cumulus cell expansion‐related genes prostaglandin‐endoperoxide synthase 2 (*PTGS2)*, pentraxin 3 (*PTX3)*, and hyaluronan synthase 2 (*HAS2)* were also significantly decreased by 0.63 ± 0.11, 0.43 ± 0.08, and 0.72 ± 0.14 times, respectively, in the si‐*TBX2* group, compared with the si‐NC group (*P* < 0.01, Figure 5c). In addition, relative cholesterol levels were reduced by 0.58 ± 0.02‐fold (*P* < 0.01), and relative lactic acid levels were reduced by 0.68 ± 0.03‐fold in the *TBX2* inhibition group compared with the si‐NC group (*P* < 0.01. Figure 5d,e).

## 4 DISCUSSION

In this study, we inhibited the expression of the *TBX2* gene to explore the physiological role of *TBX2* in bovine cumulus cells. In general, after *TBX2* was inhibited, the cell cycle was disrupted, the intracellular oxidative stress and autophagy levels were increased and the rate of cell apoptosis was also increased, suggesting that *TBX2* can regulate the physiological functions of bovine cumulus cells.

The cell cycle plays an important role in cell proliferation and apoptosis (Pietenpol & Stewart, 2002; Ye et al., 2017). Previous studies had shown that inhibition of *TBX2* resulted in an increase in G1 phase cells and a decrease in S phase cells (Pan et al., 2015; Yi et al., 2017b). In this study, changes in cyclin‐dependent kinase 2 (*CDK2)* expression may regulate the G1/S phase transition, as well as DNA synthesis and replication in S phase (Ferguson & Maller, 2010; Ohtsubo et al., 1995). However, the expression of *CDK2* did not change significantly. This may be because *TBX2* promoted cell cycle progression through cyclin D1 and retinoblastoma protein‐E2 transcription factor 1 but not *p21* and *CDK2* (Pan et al., 2015). The differential expression of *CDK1* indicates that inhibition of *TBX2* can promote entry into M phase and the transition from G2 to M phase, thus contributing to mitotic progression in cell division (Barr & Gergely, 2007; Ito, 2000). In addition, inhibition of *TBX2* may also be associated with D‐type cyclins (D1, D2, and D3) and *CDK4/6* and disrupt the essential processes for entry into G1 phase (Sherr & Roberts, 2004). In the G1 phase, *CDK4/6‐cyclin D* promotes cell cycle progression by means of retinoblastoma protein phosphorylation and sequestration of *p21* and *p27*. This indicated that inhibition of *TBX2* reduced the release of *CDK2‐cyclin E* complexes as well as *CDK2* kinase activity (Bai et al., 2017). In addition, these results are also consistent with existing reports and our subsequent findings that inhibition of *TBX2* affects cell proliferation. Changes in cell proliferative capacity are influenced by nutrient utilisation, mitochondrial function, and the physiological state of cells, which ultimately affect the biological functions of cells (Mandal et al., 2011). Combined with the results of previous studies, our findings suggest that *TBX2* may have a potential regulatory effect on the physiological and secretory functions of cumulus cells (Fu, Chen, Li, et al., 2019).

Apoptosis can also be initiated by decreased mitochondrial activity and ROS‐induced oxidative stress in addition to abnormal changes in the cell cycle (Atsumi et al., 2006). In this study, inhibition of *TBX2* significantly increased *BAX/BCL2* levels and the apoptosis rate. This is in accordance with the findings of another study in which *TBX2* overexpression reduced *caspase 3* cleavage and induced *BCL2* upregulation (Yi et al., 2017a). In addition, ROS accumulation is associated with mitochondrial fission and affects cell proliferation and apoptosis (Cadenas, 2018; Wang et al., 2017; Zorov et al., 2014). Given the MMP and ROS assay results, we suspect that inhibition of *TBX2* disrupts the mitochondrial fission/fusion balance in cumulus cells (Yi et al., 2017b), which results in an increase in intracellular ROS levels and a decrease in mitochondrial function. Subsequently, the level of autophagy can be upregulated by cells to adapt to the adverse internal environment (Twig et al., 2008), which indicates that the level of *TBX2* is important for maintaining environmental stability and generating the building blocks necessary for macromolecular synthesis, energy production, and cell survival (Lee et al., 2014).

Finally, we examined the potential effects of *TBX2* inhibition on the expansion and physiological function of cumulus cells. The expansion of cumulus cells is closely related to the development and maturation of oocytes (Su et al., 2009). In this study, inhibition of *TBX2* reduced cumulus cell expansion, consistent with the observed downregulation of important genes affecting cumulus cell expansion, such as *PTX3*, *PTGS2*, and *HAS2* (Fang et al., 2019; Kahraman et al., 2018). Previous studies showed that oocytes are unable to synthesise cholesterol and require cumulus cells to provide products of the cholesterol biosynthetic pathway (Payne & Hales, 2004; Su et al., 2008). The expansion of cumulus cells has a substantial regulatory effect on oocyte secretion, which is associated with metabolic processes in COCs, especially cholesterogenesis (Su et al., 2009; Sugiura et al., 2007). In this study, inhibition of *TBX2* led to a decrease in the cholesterol level, which indicates that inhibition of *TBX2* will lead to a decrease in precursor substances for the synthesis of steroid hormones (Miller, 2017). This also suggests that *TBX2* may have a regulatory effect on the secretory function of cumulus cells and affect oocyte maturation and fertilisation (Bunel et al., 2014; Watanabe et al., 2013). Therefore, we hypothesised that *TBX2* would potentially regulate the cumulus cell contact and secretory function that will have a definite impact on the cumulus–oocyte microenvironment (Diogenes et al., 2017), thereby playing a potential role in oocyte development, maturation, and subsequent fertilisation.

## 5 CONCLUSION

In conclusion, the results of this study showed that *TBX2* plays important roles in regulating cell proliferation, expansion, apoptosis and autophagy and maintaining the mitochondrial function and cholesterol generation of bovine cumulus cells.

## AUTHOR CONTRIBUTIONS

*Data curation, formal analysis, investigation, visualisation, writing—original draft, writing—review and editing*: Sheng‐Peng Li. *Funding acquisition, project administration, supervision, writing—original draft, writing—review and editing*: Hao Jiang. *Data curation, formal analysis, investigation*: Zi‐Bin Liu. *Formal analysis, investigation*: Wen‐Jie Yu. *Investigation, visualisation*: Xiao‐Shi Cai. *Investigation, resources, visualisation*: Chang Liu. *Investigation*: Wen‐Yin Xie. *Methodology*: Fu‐shi Quan. *Methodology*: Wei Gao. *Project administration, visualisation*: Bao Yuan. *Conceptualisation, resources, validation*: Cheng‐Zhen Chen. *Funding acquisition, investigation, resources, supervision*: Jia‐Bao Zhang.

## CONFLICT OF INTEREST

The authors declare that they have no conflict of interest.

### ETHICS STATEMENT

Animal experiments are conducted under the supervision of the Laboratory Animal Ethics and Welfare Committee (IACUC) of Jilin University, and comply with the requirements of Jilin University's laboratory animal ethics and welfare.

### PEER REVIEW

I would not like my name to appear with my report on Publons https://publons.com/publon/10.1002/vms3.1009.

## Supporting information



TABLE S1 Primer and siRNA sequences used in this study
FIGURE S1 Expression changes of TBX2 after siRNA transfection

## Data Availability

The data that support the findings of this study are available from the corresponding author upon reasonable request.
