# Supplementary material for: TBX2 affects proliferation, apoptosis and cholesterol generation by regulating mitochondrial function and autophagy in bovine cumulus cell
Source: Vet Med Sci. 2022 Nov 29;9(1):326–35. doi: 10.1002/vms3.1009 (PMC9857127; doi:10.1002/vms3.1009)
Supplement: Supplementary file 1 — TABLE S1 Primer and siRNA sequences used in this study FIGURE S1 Expression changes of TBX2 after siRNA transfection [file VMS3-9-326-s001.docx]

**Supplementary Table S1. Primer and siRNA sequences used in this study**

| **Primers/siRNA**  **name^a^** | **Sequence**  **(5' to 3')^b^** | **Product size** | **GenBank accession**  **Number** |
| --- | --- | --- | --- |
| *β-actin* | F:TCCTGTGGCATCCACGAAACT | 112 bp | NM_001206502.1 |
|  | R:GAAGCATTTGCGGTGGACGAT |  |  |
| *TBX2* | F:CTTGCAGTGCTCCTCCTA | 158 bp | NM_005994.4 |
|  | R:CACGCAGCTTA GATCGACA |  |  |
| *BCL2* | F:TGGCCTTCTTTGAGTTCGGA | 181 bp | NM_214285 |
|  | R:GGCCATACAGCTCCACAAAG |  |  |
| *BAX* | F:GGCGGCTGAAATGTTTTCTGA | 193 bp | NM_000633.3 |
|  | R:GTCCAATGTCCAGCCCATGAT |  |  |
| *CDK1* | F:CATTCGCCGCGGATAAAGC | 152 bp | NM_001098958.1 |
|  | R:CCACTTGGCCTGTAGTTTTGTG |  |  |
| *CDK2* | F:GAGCCTCGGTTGCATCTTTG | 131 bp | NM_001014934.1 |
|  | R:CCACTTGGGGAAACTTGGCT |  |  |
| *CDK4* | F:AAGGTGACGCAGAGTGAACA | 123 bp | NM_001037594.1 |
|  | R:GAGATGAAGGCAGAGGTTGC |  |  |
| *CDK6* | F:GCCTTGCTCGCATCTACAGT | 247 bp | NM_001145306.2 |
|  | R:GTCGACATCTGAACTTCCACGG |  |  |
| *PTGS2* | F:GCTGTACGTAGTCTTCAATCACAATC | 279 bp | NM_000963.4 |
|  | R:CTTAAACAAGAGCATCCAGAATGG |  |  |
| *PTX3* | F:GCTTGTCCCACTCGGAGTTC | 225 bp | NM_002852.4 |
|  | R:CATGTATGTGAATTTGGACAACGA |  |  |
| *HAS2* | F:ACACAGACAGGCTGAGGACAACTT | 407 bp | NM_005328.3 |
|  | R:AAGCAGCTGTGATTCCAAGGAGGA |  |  |
| TBX2-siRNA-685 | Sense-GGACAUUGUGGCCGCUGAU***TT*** | | |
|  | Antisense-AUCAGCGGCCACAAUCUCC***TT*** | | |
| TBX2-siRNA-708 | Sense-CCGGUAUAAAUUCCAUAA***TT*** | | |
|  | Antisense-UUAUGGAAUUUAUACCGGC***TT*** | | |
| NC-siRNA | Sense-UUCUCCGAACGUGUCACGU***TT*** | | |
|  | Antisense-ACGUGACACGUUCGGAGAA***TT*** | | |

^a^ F: Forward primer; R: Reverse primer.

^b^ Additional “***TT***” was added to 3’-end region to enhance the silencing effect and the stability of siRNA.


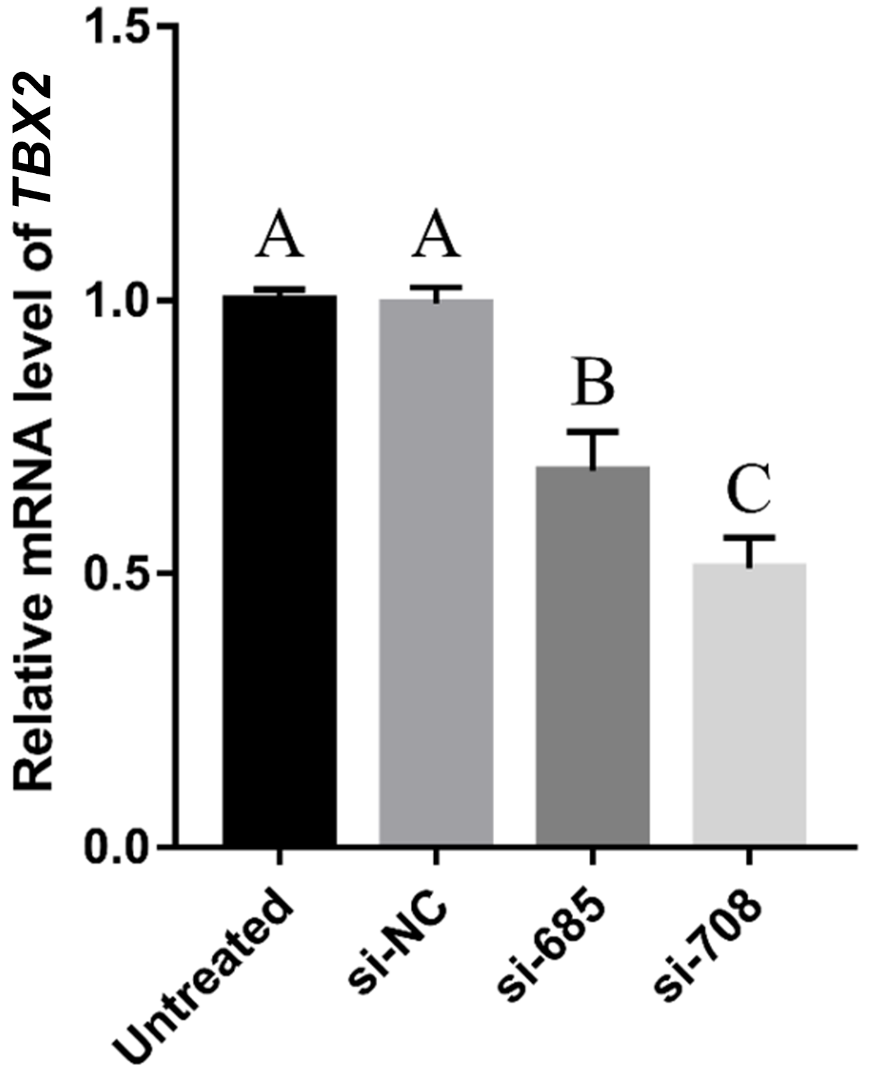


**Supplementary Figure S1. Expression changes of T‐box transcription factor 2 (*TBX2*) after siRNA transfection.**

The mRNA levels of *TBX2* with different siRNA treatment were determined by quantitative real-time reverse transcription polymerase chain reaction (qRT-PCR). Significant differences are represented by different capital letters (*P* < 0.01).
